# Myocardial Infarction in Centenarians. Data from The Polish Registry of Acute Coronary Syndromes

**DOI:** 10.3390/jcm9103377

**Published:** 2020-10-21

**Authors:** Jacek Piegza, Lech Poloński, Aneta Desperak, Andrzej Wester, Marianna Janion, Wiesław Mazurek, Wojciech Wojakowski, Adam Witkowski, Dariusz Dudek, Mariusz Gąsior

**Affiliations:** 1 Third Department of Cardiology, Faculty of Medical Sciences in Zabrze, Medical University of Silesia, 40-055 Katowice, Poland; kardiologiazabrze@sum.edu.pl (L.P.); acis777@gmail.com (A.D.); m.gasior@op.pl (M.G.); 2 Department of Physiology, Institute of Medical Sciences, University of Opole, 45-040 Opole, Poland; andrzej.wester@neostrada.pl; 3 Cardiology Center Kluczbork SCANMED, 46-200 Kluczbork, Poland; 4 Collegium Medicum, Jan Kochanowski University, 25-369 Kielce, Poland; mjanion@interia.pl; 5 Department of Cardiology, The Ludwik Rydygier Provincial Polyclinical Hospital, 87-100 Torun, Poland; wiemaz62@o2.pl; 6 3rd Division of Cardiology and Structural Heart Diseases, Medical University of Silesia, Faculty of Medical Sciences in Katowice, 40-055 Katowice, Poland; wojtek.wojakowski@gmail.com; 7 Department of Interventional Cardiology and Angiology, National Institute of Cardiology, 04-628 Warsaw, Poland; witkowski@hbz.pl; 8 Department of Interventional Cardiology, Jagiellonian University, 31-007 Krakow, Poland; mcdudek@cyfronet.pl

**Keywords:** myocardial infarction, elderly, centenarians, percutaneous coronary intervention

## Abstract

Background: There are no data regarding the mortality rate, risks and benefits of particular reperfusion methods and pharmacological treatment complications in patients aged over 100 years with acute coronary syndromes. We sought to assess the treatment of myocardial infarction (MI) in patients older than 100 years and to determine prognostic factors for this group. Methods: Among the 716,566 patients recorded between 2003 and 2018 in the Polish Registry of Acute Coronary Syndromes, 104 patients aged ≥100 with MI were included. The patients were categorized into two groups: group 1 received conservative treatment (64 patients), and group 2 received invasive strategy (40 patients). Results: The frequencies of in-hospital mortality, MI and stroke were similar in both arms. No difference in the frequency of the combined endpoint (death, reinfarction, stroke) was noted. Invasive treatment was more advantageous for 12-month outcomes; 50 patients in group 1 (79%) and 23 patients in group 2 (57.50%) died (*p* = 0.017). The multivariate analysis identified the lower left ventricular ejection fraction (EF) (Hazard Ratio (HR) = 0.96; 95% Confidence Interval (CI): 0.94–0.99; *p* = 0.012), lack of coronary angiography (HR = 0.49; 95% CI: 0.24–0.99; *p* = 0.048) and cardiac arrest (HR = 4.61; 95% CI: 1.64–12.99; *p* = 0.0038) as predictors of 12-month mortality in this group. Conclusions: Invasive MI treatment may be beneficial for selected very old patients.

## 1. Introduction

The percentage of elderly patients in the population of patients hospitalized with acute coronary syndromes (ACSs) has grown. The insufficient representation of this group in randomized studies over the years has resulted in deficient knowledge regarding the optimal treatments for this age group. The American College of Cardiology/American Heart Association (ACC/AHA) 2014 guidelines refer to patients aged above 75 as “elderly” and patients older than 90 years as “very old” [1]. The current guidelines for the management of ACSs in elderly patients without persistent ST-segment elevation recommend considering the same diagnostic and invasive strategy as for younger patients [2]. The guidelines for the management of ST-elevation myocardial infarction (STEMI) state that there is no upper age limit concerning reperfusion. The guidelines emphasize the difficulties in diagnosing MI in such patients; these difficulties result in delayed treatment and in inappropriate dosing of medicines, particularly antithrombotic therapies [3].

The preferred reperfusion method in patients presenting with ST-elevation myocardial infarction (STEMI) is primary angioplasty. Senior primary angioplasty in MI (PAMI) demonstrated that in STEMI patients aged 70–80 years, the combined endpoints (death, disabling stroke or reinfarction) occurred within 30 days after myocardial infarction (MI) more often in patients treated with thrombolytic therapy than in patients treated with angioplasty. The efficacy of both reperfusion methods was the same in the oldest age group (>80 years) [4]. The prospective randomized Tratamiento del Infarto Agudo de Miocardio en Ancianos (TRIANA) trial did not demonstrate an advantage of one method over the other in patients with a mean age of 81 years [5].

MI patients aged over 100 years are being increasingly admitted to cardiology wards. There are no data in the literature on the mortality rate, risks and benefits of particular reperfusion methods and pharmacological treatment complications apart from a general conclusion that this group is a high-risk group of patients. Moreover, it is unknown whether reperfusion therapy in elderly patients is justified in beneficial.

The goal of this paper was to assess the treatment of MI in patients older than 100 years and to determine prognostic factors for this group of patients.

## 2. Methods

### 2.1. Study Design

We used data from the Polish Registry of Acute Coronary Syndromes (PL-ACS) registry, which is a nationwide, multicenter, prospective registry of consecutively hospitalized patients with ACS in Poland. The registry is a joint initiative of the Silesian Cener for Heart Diseases Zabrze and the Polish Ministry of Health. Logistic support is provided by the National Health Fund (NHF), which is the nationwide, obligatory public health insurance institution in Poland. A detailed protocol with inclusion and exclusion criteria, methods and logistics and definitions of all the fields in the registry dataset was precisely developed before the registry was launched. Patients were continuously enrolled after a diagnosis of ACS was confirmed.

The collected data for analysis included the hospitalization period and the 30-day, 6-month and 12-month mortality rates. The posthospitalization data were obtained from official NHF data, ensuring complete information.

**Figure 1:**
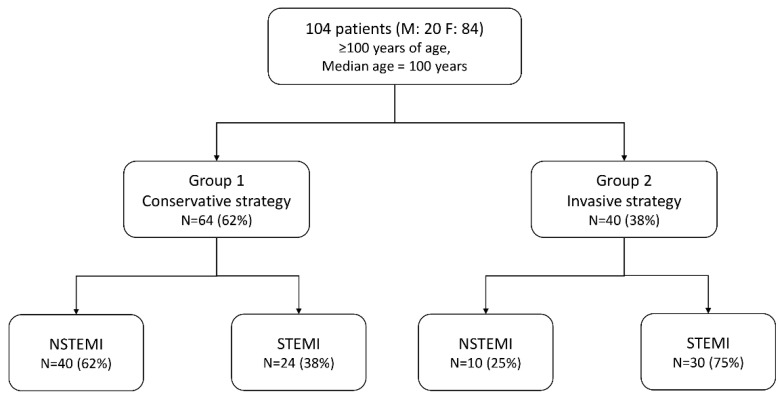
Study design. F = females; M = males; NSTEMI = non-ST-elevation myocardial infarction; STEMI = ST-elevation myocardial infarction.

The patients were categorized into two groups: group 1, patients who received conservative treatment and group 2, patients who received invasive strategy (Figure 1).

### 2.2. Definitions

The primary outcome measure was death for all-cause at 12 months. In-hospital death for all-cause, nonfatal MI, stroke and a combined endpoint (in-hospital death for all-cause, nonfatal MI and/or stroke) were considered as secondary outcome measures.

Invasive strategy was the performance of coronary angiography for MI. Conservative treatment was defined as no coronary angiography.

MI, STEMI and non-ST-elevation MI (NSTEMI) were defined according to contemporary definitions [2,3].

Stroke was defined as an ischemic event that was in accordance with the contemporary European Stroke Organisation guidelines [6].

### 2.3. Statistical Analysis

The analysis was carried out for individual groups depending on whether an invasive or conservative treatment strategy was applied. Qualitative data were analyzed using Yates’ chi-squared test. The results are presented as percentages. The analysis of normally distributed quantitative data was conducted with Student’s t-test. The data are presented as means and standard deviations. For non-normal distributions, data were compared using the Mann–Whitney U test; quantitative data are presented as medians with first and quartiles (Q1–Q3). The survival analysis was based on the Kaplan–Meier method. Factors influencing the 12-month mortality rate were analyzed using a Cox proportional regression model. As a first step, the univariate analysis of candidate variables was performed. The statistical significance level was set to *p* < 0.05. Then, a multivariate analysis, including variables with *p* < 0.10 at univariate analysis for prognosis, was performed by stepwise backward elimination. The results are presented as hazard ratios (HRs) and 95% confidence intervals (CIs). The statistical significance level was set to *p* < 0.05. All calculations were made in STATISTICA 13 (Statsoft, Inc., Tulsa, OK, USA).

## 3. Results

### 3.1. Baseline Characteristics

A total of 716,566 patients with ACSs were recorded in the PL-ACS from 2003–2018, including 438,810 patients with MI (both STEMI and NSTEMI). The group included 104 patients aged ≥100 years (100–107 years, median 100 years; 0.02% of the registered population). Twelve-month follow-up data were available for 103 out of 104 patients.

Group 1 consisted of 64 patients (61.5%) and group 2 consisted of 40 patients (38.5%). STEMI was diagnosed in 54 patients (52%), while NSTEMI was diagnosed in 50 patients (48%). All patients eligible for invasive treatment underwent coronary angiography. The procedure ended with angioplasty in 31 patients (77.5%). Acetylsalicylic acid was used in 90.5% of the patients in group 1 and in 95% of the patients in group 2, while clopidogrel was used in 65.5% and 90% of the patients, respectively. The basic clinical data of the patients with conservative and invasive treatments are shown in Table 1. The groups were homogeneous in terms of age, frequency of diabetes and history of MI. The conservative treatment group had an increased number of patients with pulmonary edema on admission and history of stroke (Table 1). The EF on admission was 40% (30–50%) in group 1 and 50% (35–50%) in group 2 (*p* = 0.30). The systolic blood pressure values were 137.5 (112–150.5) and 127.5 (110–161), respectively, while the diastolic blood pressure values were 80 mmHg (70–90) and 72.5 mmHg (70–80), respectively. In 34 (85%) invasive-strategy patients, IRA (infarct-related artery) was ascertained. Among them, 91.2% were performed PCI (percutaneous coronary intervention) of IRA.

### 3.2. In-Hospital and Follow-Up Outcome

Table 2 shows the primary complications during hospitalization. Table 3 shows the 30-day, 6-month and 12-month mortality rates.

The frequencies of in-hospital death, MI and stroke were similar in both arms. No difference in the frequency of the combined endpoint (death, reinfarction or stroke) was observed.

**Figure 2:**
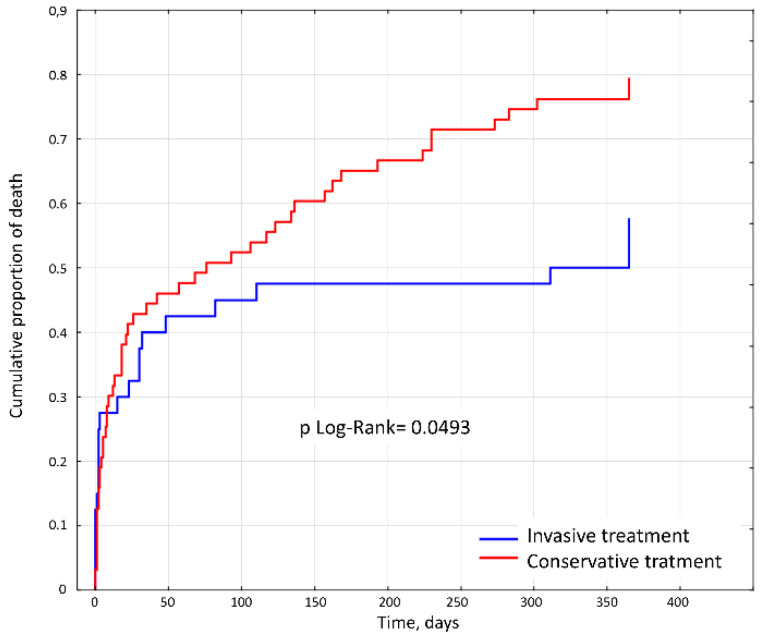
Kaplan–Meier survival curves for 12-month rates of all-cause mortality for the study population.

The 30-day and 6-month mortality rates were similar in both groups. Invasive strategy was more advantageous for 12-month outcomes; 50 patients in group 1 (79%) and 23 patients in group 2 (57.50%) died (*p* = 0.017). The Kaplan–Meier plots are shown in Figure 2.

According to a multivariate analysis, an increased EF and coronary angiography improved the prognosis, while cardiac arrest worsened the prognosis (Table 4).

### 3.3. Comparison to PL-ACS Population

A comparative analysis of the overall group with the other patients in the registry demonstrated that patients older than 100 years suffered less often from diabetes (16.67% vs. 25.28%) and hypertension (60.58% vs. 69.65%) than patients younger than 100 years. The analyzed centenarians exhibited a lower frequency of hypercholesterolemia (19.61% vs. 40.56%), history of MI (10.58% vs. 19.32%) and obesity (3.85% vs. 19.83%) than the remaining population. They were diagnosed with chronic kidney disease (80.3% vs. 7.5%) more often than the remaining population.

### 3.4. STEMI Patients

There were no differences in baseline clinical characteristics between conservative and interventional strategies in STEMI patients. No statistically significant differences regarding 30-day-, 6-month- and one-year mortality were ascertained (Table 5).

## 4. Discussion

### 4.1. Myocardial Infarction in Centenarians

There are few data on the frequency and course of ACS in the oldest age groups. This fact is significant, as approximately 60% of ACS admissions involve patients older than 65 years, and 85% of ACS-related deaths occur in this age group. More hospitalizations due to NSTEMI (approximately 32–43%) than due to STEMI (24–28%) are observed in the oldest patients [7,8,9]. In 2009, 40.6% of patients hospitalized with MI (both STEMI and NSTEMI) were older than 75 years, according to a large German national registry. The in-hospital mortality rate in the group older than 95 years was 46% in the case of STEMI and 30% in the case of NSTEMI [10]. In 2012, 35% of patients hospitalized with MI (both STEMI and NSTEMI) were older than 75 years, and 10% were older than 85 years in Poland [11]. The annual mortality rate in the oldest age group (≥80 years) was 24.8%, while in the group younger than 64 years, it was 4.1% [11]. In addition, older patients (>90 years) also have a higher rate of cardiac tamponade, cardiogenic shock after PCI and bleeding complications requiring blood transfusion [12]. The risk of death is assumed to increase by 75% with every ten years of age [13]. The percentage of heart and vascular diseases in the 75+ years population increased over ten years (2000–2010) from 30.8 to 35.5% [3]. Despite the fact that ACS affects mainly elderly individuals, in clinical trials, only 10% of patients were aged 75 years and older, and less than 2% of patients were older than 85 years [14]. There is no literature on the management of MI in patients older than 100 years. We identified 104 centenarians in the PL-ACS, which was 0.02% of the whole registered population.

### 4.2. Clinical Symptoms

The reason for hospitalization in most of the patients in the cohort (over 72%) was chest pain. These results did not conform to the data in the literature. According to many studies, including the National Registry of MI (NRMI), pain is the primary sign in only 40% of patients older than 85 years; dyspnea is much more common than pain [8,15]. This discrepancy may result from a significant age difference between the two analyzed populations or from the fact that aggravating factors, such as diabetes, were identified in this population less often than in the whole population of MI patients [16].

### 4.3. Comorbidities

The frequency of some other diseases was lower in the oldest group than in the whole population in the PL-ACS registry (Table 6). Renal diseases were diagnosed in a greater proportion of patients aged more than 100 years than in the whole population presenting with MI, while hypertension was diagnosed in the centenarian group only slightly less often than in the whole population. Diabetes, a history of MI and hypercholesterolemia were much less common than renal diseases. A low proportion of patients smoked. Other authors noted similar observations and stressed the importance of genetic profiles, healthy lifestyles and consistent treatment for very old patients [17,18,19,20].

### 4.4. Sex-Specific Data

Contrary to popular belief, the oldest age group is not more burdened than patients older than 75 years. The present group was dominated by women, which is in line with the general life expectancy trends for women and men. The Nationwide Acute Myocardial Infarction (AMI-PL) registry is dominated by men, as it includes mostly relatively young patients with MI. The share of women and men equalized in the 75–79-year interval. Women were hospitalized twice as often as men in the older than 85 years group [11]. The increase in women with time accounts for their significant proportion in the investigated population. This result is primarily related to the fact that women tend to live longer. In Poland, women live for an average of 82 years, whereas men live for an average of 74 years [21].

### 4.5. Invasive Treatment

Invasive treatment was more common in STEMI patients than in NSTEMI patients. Administering conservative treatment as opposed to the implementation of invasive treatment was undoubtedly due to the aggravating factor of age and the increased risks of hemorrhagic and thrombotic complications. The present study, as well as the randomized After Eighty study, failed to confirm the concerns reported in the literature [22]. The After Eighty study involved 457 patients aged 80+ years (mean age slightly less than 85 years) with ST-segment elevation ACSs. The advantage of the invasive strategy over conservative treatment was demonstrated, with no increase in the risk of hemorrhagic complications. No similar data for STEMI patients are available. AMI-PL data in 2009 show that MI angioplasty was performed in 36% of patients older than 80 years in Poland. In the whole population, invasive treatment was more popular in patients with STEMI (67.7%) than in those with NSTEMI (47.9%) [11].

### 4.6. In-Hospital and Follow-Up Mortality

The mortality rate for centenarians presenting with MI is high. The in-hospital mortality rate was nearly 35%, the 30-day mortality rate was 45%, the 6-month mortality rate was 64%, and the 12-month mortality rate was 70%. The German registry recorded the hospital mortality rate for those above the age of 75 years as 17.2%, while for the oldest population (above 95 years), the mortality rates were 45% for STEMI patients and 30% for NSTEMI patients. The in-hospital mortality rate for patients with MI who were aged 80+ years was 20% in 2009 and 17.1% in 2012, according to the AMI-PL [11]. A Danish study on two cohorts of patients, those 80+ years and 90+ years, found that the 30-day mortality rates for primary percutaneous coronary intervention (PCI) STEMI patients was 17.2% for patients 80+ and 25.8% for patients 90+. The 12-month mortality rates were 27.6% and 32.5%, respectively. If we assume a 75% mortality rate increase per 10 years of life, our results are similar to the findings of the Danish study [23]. Even though differences in 12-month mortality have been shown both in STEMI and NSTEMI patients, in the STEMI patients, no such differences were ascertained. The fact that there were few STEMI patients may have induced the lack of statistically significant differences.

The results show that in the long term, intervention therapy may be beneficial for the whole group of patients with MI. The potential benefits of intervention therapy are most likely offset by complications occurring in the first months after angioplasty. The most notable complications seem to be hemorrhagic and ischemic complications and complications related to the use of contrast agents in patients with kidney damage. After a few months, the favorable effect of improved perfusion likely starts to dominate, resulting in improved function of the left ventricle and a reduced risk of heart arrhythmia (Figure 2). According to the multivariate analysis, coronary angiography may be a positive prognosis factor similar to a high EF (Table 4). The efficacy of angioplasty and thrombolytic therapy as methods of managing MI in elderly and very old patients has been compared in many papers; these papers have indicated that the former is a better method. There are no articles, however, comparing angioplasty with conservative treatment in the oldest age group apart from a Norwegian publication on non-ST-segment elevation ACS (NSTE-ACS) patients [21]. A literature analysis demonstrated that the efficacy of MI intervention is greater than that of conservative management in all age groups despite its associated complications. Our study shows that this is also true for patients with MI aged 100 years and older.

### 4.7. Study Limitations

This study has several limitations. The PL-ACS was a voluntary, observational registry, and not all hospitals treating infarctions in Poland participated in data collection. The present analysis was retrospective, and some potentially important parameters may not be included. The data on mortality were obtained from the NHF, which is the most reliable data source. However, NHF does not provide details on deaths’ causes. Even though we had detailed baseline data, we had no information on some basic background like physical activity, presence of dementia or family support. These factors might have had an impact on strategy choice and, in consequence, affect the outcomes of the centenarians. Moreover, some of the significant study limitations included lack of data concerning the anatomy of coronary lesions (i.e., chronic total occlusion, diameter stenosis, calcifications) and the cause of not performing PCI in patients from the group with an invasive strategy.

Finally, this was a single-country study; therefore, some trends should be interpreted with caution.

## 5. Conclusions

The selection of MI treatments in centenarians must be considered on an individual basis; one criterion, aside from the condition of the patient, should be the experience of the specific organization. It seems that the invasive method should be conducted more often, particularly in STEMI patients and some specific NSTEMI patients. Aggravating factors need to be taken into consideration in each case. Despite the high mortality rate, invasive strategy is more effective than a conservative approach. However, all-cause mortality in centenarians may be influenced by many comorbidities that may result in death. Invasive treatment should be considered in each case and should be rejected in only very justified cases. Invasive MI treatment may be beneficial for selected very old patients.

## Figures and Tables

**Table 1:** Baseline clinical characteristics.

Factor	Group 1 *n* = 64 (61.5%)	Group 2 *n* = 40 (38.6%)	*p*
Age, Years (Q1–Q3)	100 (100–101)	100 (100–102.5)	0.77
Male, *n* (%)	11 (17.9)	9 (22.50)	0.50
Direct Admission, *n* (%)	58 (90.63)	25 (62.50)	0.0005
Chest pain, *n* (%)	17 (62.96)	31 (79.49)	0.14
EF, % (Q1–Q3)	40 (30–50)	50 (35–50)	0.30
Sinus Rhythm, *n* (%)	43 (67.19)	29 (72.50)	0.57
Pulmonary Edema, *n* (%)	12 (18.75)	2 (5.00)	0.046
Cardiogenic Shock, *n* (%)	2 (3.13)	2 (5.00)	0.63
STEMI, *n* (%)	24 (37.50)	30 (75.00)	0.0002
Arterial Hypertension, *n* (%)	34 (53.13)	29 (72.50)	0.049
Hypercholesterolemia, *n* (%)	12 (19.05)	8 (20.51)	0.86
Diabetes Mellitus, *n* (%)	11 (17.19)	6 (15.79)	0.85
Cigarette Smoking, *n* (%)	3 (4.76)	5 (12.50)	0.15
Obesity, *n* (%)	3 (4.69)	1 (2.50)	0.57
History of MI, *n* (%)	7 (10.94)	4 (10.00)	0.88
History of Stroke, *n* (%)	3 (11.11)	0 (0.00)	0.033
PAD, *n* (%)	2 (7.41)	0 (0.00)	0.08
Kidney Disease, *n* (%)	22 (81.48)	31 (79.49)	0.84
Prior PCI, *n* (%)	0 (0.00)	2 (5.13)	0.07

EF = left ventricular ejection fraction, MI = myocardial infarction, PAD = peripheral artery disease, PCI = percutaneous coronary intervention, STEMI = ST-elevation myocardial infarction.

**Table 2:** Management and in-hospital outcome.

Factor	Group 1 *n* = 64 (61.5%)	Group 2 *n* = 40 (38.6%)	*p*
PCI, *n* (%)	0 (0.00)	31 (77.50)	<0.0001
Aspirin on Discharge, *n* (%)	58 (90.63)	38 (95.00)	0.42
Clopidogrel on Discharge, *n* (%)	42 (65.63)	36 (90.00)	0.0052
All-Cause Mortality, *n* (%)	24 (37.50)	12 (30.00)	0.43
MI, *n* (%)	1 (1.56)	0 (0.00)	0.43
Stroke, *n* (%)	2 (3.13)	0 (0.00)	0.26
Cardiac Arrest, *n* (%)	12 (18.75)	8 (20.00)	0.87
Death, Stroke or MI, *n* (%)	25 (39.06)	12 (30.00)	0.35

MI = myocardial infarction, PCI = percutaneous coronary intervention.

**Table 3:** Management and in-hospital outcome.

Factor	Group 1 *n* = 64 (61.5%)	Group 2 *n* = 40 (38.6%)	*p*
30-Day Mortality, *n* (%)	31 (48.44)	16 (40.00)	0.40
6-Month Mortality, *n* (%)	45 (70.31)	22 (55.00)	0.11
One-Year Mortality, *n* (%)	50 (79.37)	23 (57.50)	0.017

**Table 4:** Univariate and multivariate analysis.

	Univariate		Multivariate	
	HR (95% CI)	*p*	HR (95% CI)	*p*
Invasive Strategy	0.62 (0.38–1.01)	0.057	0.49 (0.24–0.99)	0.048
SBP, per 10 mmHg more	0.90 (0.83–0.96)	0.0014	1.01 (0.89–1.15)	0.87
EF, per 10% more	0.55 (0.43–0.70)	<0.0001	0.70 (0.53–0.92)	0.012
Cardiac Arrest	5.09 (2.96–8.76)	<0.0001	4.61 (1.64–12.99)	0.0038

Candidate variables for the model: age, left ventricular ejection fraction, systolic and diastolic blood pressure, sinus rhythm on admission, chronic kidney disease, pulmonary disease, diabetes mellitus, in-hospital cardiac arrest, STEMI diagnosis; CI = confidence interval, EF = left ventricular ejection fraction, HR = hazard ratio, SBP = systolic blood pressure.

**Table 5:** Mortality in STEMI population.

Factor	Group 1 *n* = 24 (44.4%)	Group 2 *n* = 30 (55.6%)	*p*
30-day Mortality, *n* (%)	54.17% (13)	46.67% (14)	0.5839
6-month Mortality, *n* (%)	79.17% (19)	66.67% (20)	0.3082
One-year Mortality, *n* (%)	83.33% (20)	66.67% (20)	0.1649

**Table 6:** Comparison of the study population (≥100 years old) and the whole Polish Registry of Acute Coronary Syndromes (PL-ACS) population.

Factor	Study Population	PL-ACS	*p*
Female, *n* (%)	80 (76.92)	159,318 (36.31)	<0.001
Arterial Hypertension, *n* (%)	63 (60.58)	305,607 (69.65)	0.044
Diabetes Mellitus, *n* (%)	17 (16.67)	110,813 (25.28)	0.045
Hypercholesterolemia, *n* (%)	20 (19.61)	177,793 (40.56)	<0.001
Cigarette Smoking, *n* (%)	8 (7.66)	215,039 (49.05)	<0.001
History of MI, *n* (%)	11 (10.58)	84,744 (19.32)	0.024
History of Stroke, *n* (%)	3 (6.06)	13,067 (4.37)	0.573
Kidney Disease, *n* (%)	53 (80.30)	22,460 (7.50)	<0.001
Prior PCI, *n* (%)	2 (1.92)	47,258 (10.78)	0.004
Obesity, *n* (%)	4 (3.85)	86,907 (19.83)	<0.001
In-Hospital Mortality, *n* (%)	36 (34.62)	16,907 (3.78)	<0.001
30-Day Mortality, *n* (%)	47 (45.19)	23,599 (4.95)	<0.001
6-Month Mortality, *n* (%)	67 (64.42)	44,871 (10.04)	<0.001
One-Year Mortality, *n* (%)	73 (70.87)	68,731 (14.48)	<0.001

MI = myocardial infarction, PCI = percutaneous coronary intervention.

## References

[1] Amsterdam E.A., Wenger N.K., Brindis R.G., Casey D.E., Ganiats T.G., Holmes D.R., Jaffe A.S., Jneid H., Kelly R.F., Kontos M.C. (2014). 2014 AHA/ACC Guideline for the Management of Patients with Non-ST-Elevation Acute Coronary Syndromes: A report of the American College of Cardiology/American Heart Association Task Force on Practice Guidelines. J. Am. Coll. Cardiol..

[2] Collet J.P., Thiele H., Barbato E., Barthélémy O., Bauersachs J., Bhatt D.L., Dendale P., Dorobantu M., Edvardsen T., Folliguet T. (2020). 2020 ESC Guidelines for the management of acute coronary syndromes in patients presenting without persistent ST-segment elevation. Eur. Heart J..

[3] Ibanez B., James S., Agewall S., Antunes M.J., Bucciarelli-Ducci C., Bueno H., Caforio A.L., Crea F., Goudevenos J.A., Halvorsen S. (2018). 2017 ESC Guidelines for the management of acute myocardial infarction in patients presenting with ST-segment elevation: The Task Force for the management of acute myocardial infarction in patients presenting with ST-segment elevation of the European Society of Cardiology (ESC). Eur. Heart J..

[4] Grines C. Senior PAMI: A prospective randomized trial of primary angioplasty and thrombolytic therapy in elderly patients with acute myocardial infarction. Proceedings of the Transcatheter Cardiovascular Therapeutics 2005.

[5] Bueno H., Betriu A., Heras M., Alonso J.J., Cequier A., García E.J., López-Sendón J.L., Macaya C., Hernández-Antolín R. (2011). TRIANA Investigators: Primary angioplasty vs. fibrinolysis in very old patients with acute myocardial infarction: TRIANA (TRatamiento del Infarto Agudo de miocardio eN Ancianos) randomized trial and pooled analysis with previous studies. Eur. Heart J..

[6] European Stroke Organisation (ESO) Executive Committee, ESO Writing Committee (2008). Guidelines for management of ischaemic stroke and transient ischaemic attack 2008. Cerebrovasc Dis..

[7] Alexander K.P., Newby L.K., Cannon C.P., Armstrong P.W., Gibler W.B., Rich M.W., Van de Werf F., White H.D., Weaver W.D., Naylor M.D. (2007). Acute coronary care in the elderly, part I: Non-ST-segment-elevation acute coronary syndromes: A scientific statement for healthcare professionals from the American Heart Association Council on Clinical Cardiology: In collaboration with the Society of Geriatric Cardiology. Circulation.

[8] Alexander K.P., Newby L.K., Cannon C.P., Armstrong P.W., Gibler W.B., Rich M.W., Van de Werf F., White H.D., Weaver W.D., Naylor M.D. (2007). Acute coronary care in the elderly, part II: ST-segment-elevation myocardial infarction: A scientific statement for healthcare professionals from the American Heart Association Council on Clinical Cardiology: In collaboration with the Society of Geriatric Cardiology. Circulation.

[9] De Luca L., Olivari Z., Bolognese L., Lucci D., Gonzini L., Di Chiara A., Casella G., Chiarella F., Boccanelli A., Di Pasquale G. (2014). A decade of changes in clinical characteristics and management of elderly patients with non-ST elevation myocardial infarction admitted in Italian cardiac care units. Open Heart.

[10] Freisinger E., Fuerstenberg T., Malyar N.M., Wellmann J., Keil U., Breithardt G., Reinecke H. (2014). German nationwide data on current trends and management of acute myocardial infarction: Discrepancies between trials and real-life. Eur. Heart J..

[11] Gierlotka M., Zdrojewski T., Wojtyniak B., Poloński L., Stokwiszewski J., Gąsior M., Kozierkiewicz A., Kalarus Z., Wierucki Ł., Chlebus K. (2015). Incidence, treatment, in-hospital mortality and one-year outcomes of acute myocardial infarction in Poland in 2009–2012—Nationwide AMI-PL database. Kardiol. Pol..

[12] Numasawa Y., Inohara T., Ishii H., Yamaji K., Kohsaka S., Sawano M., Kodaira M., Uemura S., Kadota K., Amano T. (2019). Comparison of Outcomes After Percutaneous Coronary Intervention in Elderly Patients, Including 10,628 Nonagenarians: Insights From a Japanese Nationwide Registry (J-PCI Registry). J. Am. Heart Assoc..

[13] Dai X., Busby-Whitehead J., Alexander K.P. (2016). Acute coronary syndrome in the older adults. J. Geriatr. Cardiol..

[14] Lee P.Y., Alexander K.P., Hammill B.G., Pasquali S.K., Peterson E.D. (2001). Representation of elderly persons and women in published randomized trials of acute coronary syndromes. JAMA.

[15] Gregoratos G. (2001). Clinical manifestations of acute myocardial infarction in older patients. Am. J. Geriatr. Cardiol..

[16] Poloński L., Gasior M., Gierlotka M., Kalarus Z., Cieśliński A., Dubiel J.S., Gil R.J., Ruzyłło W., Trusz-Gluza M., Zembala M. (2007). Polish Registry of Acute Coronary Syndromes (PL-ACS): Characteristics, treatments and outcomes of patients with acute coronary syndromes in Poland. Kardiol. Pol..

[17] Bertinieri G., Grassi G., Rossi P., Meloni A., Ciampa M., Annoni G., Vergani C., Mancia G. (2002). 24-hour blood pressure profile in centenarians. J. Hypertens..

[18] Terry D.F., Wilcox M.A., McCormick M.A., Perls T.T. (2004). Cardiovascular disease delay in centenarian offspring. J. Gerontol. A Biol. Sci. Med. Sci..

[19] Suzuki M., Wilcox B.J., Wilcox C.D. (2001). Implications from and for food cultures for cardiovascular disease: Longevity. Asia Pac. J. Clin. Nutr..

[20] Barter P. (2004). HDL: A recipe for longevity. Atheroscler. Suppl..

[21] Rozkrut D., Adach-Stankiewicz E., Bielak R., Bieniek M., Błażej M., Jeznach M., Kamińska-Gawryluk E., Kursa L., Mojsiewicz M., Rogalińska M. (2017). Statistical Yearbook of the Republic of Poland.

[22] Tegn N., Abdelnoor M., Aaberge L., Endresen K., Smith P., Aakhus S., Gjertsen E., Dahl-Hofseth O., Ranhoff A.H., Gullestad L. (2016). After Eighty study investigators. Invasive versus conservative strategy in patients aged 80 years or older with non-ST-elevation myocardial infarction or unstable angina pectoris (After Eighty study): An open-label randomised controlled trial. Lancet.

[23] Antonsen L., Jensen L.O., Terkelsen C.J., Tilsted H.H., Junker A., Maeng M., Hansen K.N., Lassen J.F., Thuesen L., Thayssen P. (2013). Outcomes after primary percutaneous coronary intervention in octogenarians and nonagenarians with ST-segment elevation myocardial infarction: From the Western Denmark heart registry. Catheter. Cardiovasc. Interv..

